# Open surgical repair of complex non-Type A and non-Type B aortic dissection in a right aortic arch–case report

**DOI:** 10.3389/fcvm.2026.1784447

**Published:** 2026-10-02

**Authors:** Tailuan Peng, Mingkun Xu, Chaohu Xiang, Jun Xiang, Yong Liu

**Affiliations:** Department of Cardiovascular Surgery, Affiliated Hospital of North Sichuan Medical College, Nanchong, Sichuan, China

**Keywords:** aortic dissection, case report, endovascular repair, non-Type A and non-Type B aortic dissection, open surgery

## Abstract

**Objective:**

To report the diagnostic and treatment process of a patient with complex non-Type A and non-Type B aortic dissection, and to discuss its clinical characteristics and surgical strategy.

**Methods:**

A retrospective analysis was conducted on the clinical data of a patient with complex non-Type A and non-Type B aortic dissection.

**Results:**

After admission, the patient was diagnosed with complex non-Type A and non-Type B aortic dissection through computed tomography angiography (CTA). The aorta exhibited a right-sided arch, with the left common carotid artery originating from the ascending aorta and the aberrant left subclavian artery arising from the distal aortic arch. The right vertebral artery originated from the right-sided aortic arch. The dissection tear was located at the distal part of the aortic isthmus, approximately 2 cm in width. The dissection flap extended proximally to the distal aspect of the right subclavian artery origin and distally to the proximal aspect of the left renal artery origin. Due to the high difficulty of endovascular repair and the increased risk of long-term complications, an open surgical approach was chosen. This involved deep hypothermic circulatory arrest with elephant trunk stenting, left common carotid to left subclavian artery bypass. The patient was discharged one week after surgery in good health.

**Conclusion:**

This case illustrates that open surgical repair can be a feasible and effective option for selected patients with non-A non-B aortic dissection and complex arch anatomy that limits endovascular repair. Treatment should be individualized according to entry-tear location, arch-branch anatomy, landing-zone feasibility, malperfusion risk, and institutional expertise. Due to the special anatomical structure and high surgical difficulty, a personalized treatment plan must be developed. Open surgery still offers irreplaceable advantages in certain cases.

## 1 Introduction

Aortic dissection is the most common acute aortic syndrome in clinical practice, with an incidence rate of 2.6–3.5 cases per 100,000 person-years, and surgical treatments represents one of the most important therapeutic strategies (1, 2). Based on the 2020 Society for Vascular Surgery (SVS) and Society of Thoracic Surgeons (STS) reporting standards as well as the European update of the Stanford Classification, a new category of non-Type A and non-Type B aortic dissection was added (3). Here, we report the case of a patient with non-Type A and non-Type B aortic dissection associated with a right-sided aortic arch and an aberrant left subclavian artery. After a thorough preoperative discussion with the patient and their family regarding the risks involved, they agreed to undergo deep hypothermic circulatory arrest with elephant trunk stenting, left common carotid to left subclavian artery bypass. The patient recovered smoothly post-operatively and has since been discharged in good health.The patient consents to the disclosure of their medical case details and imaging materials.

## 2 Patient information

A 62-year-old male patient, who presented with intermittent tearing retrosternal pain that had suddenly occurred 7 days prior, was admitted to the hospital. The onset of symptoms was accompanied by profuse sweating, shortness of breath, nausea, and vomiting. Physical examination revealed no significant abnormalities: the patient was conscious, with normal muscle strength in all four limbs. The patient had been suffering from hypertension for over ten years but had not been adhering to regular antihypertensive medication. Seven years ago, he underwent lower extremity steel plate internal fixation. He has no history of diabetes, hepatitis, tuberculosis, or other infections. Upon admission, the patient's vital signs were monitored: blood pressure 158/76 mmHg, heart rate 86 beats per minute, respiratory rate 14 breaths per minute, and oxygen saturation of 99% without supplemental oxygen. To further clarify the diagnosis, a chest and abdominal vascular CTA was performed, revealing the following findings: a right-sided aortic arch, with the aortic arch, descending aorta, and abdominal aorta all showing enlargement. The dissection flap extended proximally to the distal aspect of the right subclavian artery origin and distally to the proximal aspect of the left renal artery origin. Irregular intimal flap and signs of calcified intimal tear were observed within the lumen. The enhanced scan showed a “double lumen” change, with the false lumen being large and the true lumen small. Both renal arteries originated from the true lumen. Supplementary Figures 1, 2, along with Supplementary Video 1, illustrate these findings.

The patient was taken to the operating room 2 days after admission. After successful general anesthesia, the patient was placed in a supine position. We exposed the left femoral artery and right axillary artery for cannulation prior to performing sternotomy. Arch vessels were exposed, including the right subclavian artery, right vertebral artery, right common carotid artery, left common carotid artery, and aberrant left subclavian artery. The ascending aorta was found to have a normal diameter. The aorta was a right aortic arch. The left common carotid artery arose from the ascending aorta, the aberrant left subclavian artery arising from the distal aortic arch, and the right vertebral artery arose from the right-sided aortic arch.The dissection tear was located at the distal part of the aortic isthmus. The dissection flap extended proximally to the distal aspect of the right subclavian artery origin. (see Supplementary Figure 3).

After completing preparations for extracorporeal circulation, cannulation was performed at the right axillary artery, the left femoral artery, the right atrium, and the left atrium to establish the bypass. The distal ascending aorta was occluded, and myocardial protection solution was infused at the root of the aorta. Ice slush was placed on the surface of the heart, and cardiac arrest was successfully achieved. Cannulation of the left and right common carotid arteries was performed for bilateral antegrade cerebral protection, and cooling was continued until a rectal temperature of 26 °C was achieved. The branches of the aortic arch were occluded, and circulatory arrest was initiated, systemic flow rate was reduced to 5 mL/kg/min, and the left upper extremity blood pressure was maintained at 20–25mmHg. A transverse incision was made in the descending aorta distal to the origin of the right subclavian artery, and a 26 Fr, 120 mm intraoperative stent was inserted into the true lumen of the descending aorta. The non-covered area of the intraoperative stent graft was directly and continuously anastomosed to the transverse incision of the descending aorta using a 4–0 Prolene suture. On the posterior wall, two additional mattress sutures reinforced with felt pledgets were placed intraluminally using 4–0 Prolene. After venting the aortic root, the ascending aorta was unclamped, and the heart resumed beating spontaneously. Due to the difficulty of reconstructing the aberrant left subclavian artery within the chest, the proximal end of the aberrant left subclavian artery was ligated. A horizontal incision was made in the left subclavian region, and the left axillary artery was dissected. Using a 5–0 Prolene suture, one end of a 10-mm vascular graft was anastomosed end-to-side to the left common carotid artery, and the other end was anastomosed end-to-side to the left axillary artery. After deairing both ends, the bypass graft was opened. Rewarming was performed until a rectal temperature of 36.5 °C was achieved. Following hemodynamic stabilization, cardiopulmonary bypass was discontinued, heparin was neutralized with protamine, and conventional hemostasis and chest closure were performed. Intraoperative blood loss was 600 mL, and the patient received 400 mL of fresh frozen plasma and 2 U of packed red blood cells. Cardiopulmonary bypass time was 173 min, ascending aortic cross-clamp time was 84 min, and circulatory arrest time was 19 min.

Two hours after surgery, the patient regained consciousness. Six hours post-surgery, the endotracheal tube was successfully removed, and 24 h post-surgery, the patient was transferred from the intensive care unit to a general ward. After one week of treatment, the patient was discharged. Before discharge, a follow-up chest and abdominal vascular CTA was performed. The findings included: a visible left common carotid-left subclavian artery bypass graft, with uniform filling of contrast agent within the graft. The right-sided aortic arch was noted, with a dense stent shadow in the aortic arch and descending aorta, and no obvious contrast agent leakage was observed on enhanced scanning. Below the stent, in the region corresponding to the chest and abdominal aorta (around the L1 vertebral level), an arc-shaped, slightly higher, isodense shadow was seen. On plain scan, a linear high-density shadow was visible within the lumen, and after contrast enhancement, no filling of contrast agent was seen in the false lumen. Supplementary Figures 4, 5, as well as Supplementary Video 2, illustrate these findings.

The patient was followed up via telephone at one month postoperatively. He reported that all parameters were favorable. Although he was advised to return for a follow-up CTA, he has not yet done so due to personal reasons. However, the patient stated that he is currently well and has no symptoms or discomfort.

## 3 Discussion

The treatment of aortic dissection is based on factors such as the classification of the disease, the extent of the lesion, and the patient's individual condition (4). Non-Type A and non-Type B aortic dissection is a unique clinical subtype, and its clinical progression differs from the other two types of aortic dissection. In non-A non-B aortic dissection, the intimal tear is located in a region other than the ascending aorta, with the dissection involving the aortic arch; alternatively, the tear is located in the descending aorta and extends retrogradely to but not beyond the aortic arch (5). In such patients, the anatomy of the aortic arch is complex and often accompanied by anatomical variations. Therefore, when determining the treatment strategy, it is necessary to comprehensively consider the location of the intimal tear, the direction of false lumen extension, and the involvement of important branch vessels. In previous treatment approaches (6), some researchers opted for the same medical management used in Type B aortic dissection to treat non-Type A and non-Type B aortic dissection. However, follow-up results showed poor long-term outcomes, with significantly higher mortality compared to surgical treatment. Therefore, growing clinical evidence supports early surgical intervention for non-Type A and non-Type B aortic dissection, particularly in patients with insufficient organ perfusion or a high risk of rupture (1, 4, 6, 7).

The case presented in this report involves a patient with a unique anatomical structure, including a large angle in the aortic arch and a dissection tear close to the right subclavian artery. The tear also involved the aberrant left subclavian artery's orifice, as well as the right vertebral artery located between the right subclavian artery and the right common carotid artery, with very little space between the two arteries. This anatomical variation greatly increased the difficulty of endovascular repair. We considered using a single-branch covered stent with the branch positioned at the right subclavian artery. However, due to the very short distance between the right subclavian artery and the right common carotid artery, and the presence of the right vertebral artery in between, this would have required an *in situ* fenestration. Additionally, the aberrant left subclavian artery would require further artificial vascular bypass to ensure adequate cerebral perfusion. For this patient, endovascular repair is not only technically complex and expensive, but also associated with a high incidence of long-term complications, and its reoperation rate is significantly higher than that of open surgery (8, 9). However, open surgery also has certain limitations. Open surgery causes greater trauma to the patient, requires a longer operative time, and carries the risk of complications associated with cardiopulmonary bypass. For patients with poor baseline conditions, the risk of mortality is higher. Considering that this patient is relatively young and in generally good physical condition, we ultimately opted for open surgery. This approach allowed for complete resection of the proximal dissection and closure of the false lumen, thereby preventing further vessel dilation. Additionally, during the entire surgical procedure, apart from performing a bypass for the aberrant left subclavian artery, no further intervention was needed for the other arch vessels. This significantly reduced the risk of cerebrovascular complications and preserved the natural anatomical pathway of the head and arm arteries, which is crucial for maintaining proper cerebral and upper limb hemodynamics. Postoperative imaging confirmed that the true lumen was patent, the false lumen had been completely sealed, and the blood supply to the arch branches was intact.

Based on a review of the relevant literature and our experience with this case, we believe that for patients with non-A non-B aortic dissection who have complex anatomy involving the aortic arch and a concurrent aberrant subclavian artery, open surgery can be confidently chosen if the patient is in generally good condition and can tolerate the risks of surgery. This approach is particularly advantageous when endovascular treatment carries high risk or is technically challenging. Individualized assessment of the patient's general condition, lesion characteristics, and vascular anatomy is key to selecting the appropriate surgical strategy. Although open surgery is more invasive, it offers irreplaceable value in achieving complete treatment of the lesion and reducing long-term complications. However, endovascular repair also has its unique advantages. Future multi-center studies are needed to further validate the long-term outcomes of different surgical approaches in this specific anatomical population.

## 4 Limitations

This case report has a significant limitation regarding the lack of standardized postoperative imaging follow-up. According to current guidelines, patients are recommended to undergo aortic CTA at 1, 3, 6, and 12 months postoperatively, and annually thereafter. However, our patient did not adhere to this schedule. Although we conducted a telephone follow-up at 1 month and advised the patient to return for CTA, he did not present to our institution. At 3 months, we learned that he had undergone imaging at an outside hospital, where he was told that the surgical results were satisfactory, however, we were unable to obtain or review those external imaging records. Consequently, objective assessment of long-term repair durability and potential late complications remains incomplete. Future studies should emphasize the importance of standardized imaging surveillance and patient compliance to better evaluate the outcomes of open surgical repair in similar complex cases.

## 5 Patient perspective

The patient expressed great satisfaction with this treatment, noting that their physical functions have now returned to normal. They extend their gratitude to the medical staff for their attentive care.

## Data Availability

The original contributions presented in the study are included in the article/Supplementary Material, further inquiries can be directed to the corresponding author.

## Supplementary material

Supplementary Figure 1 Preoperative Chest and Abdominal Vascular CTA. The left image shows the dissection involving the distal right subclavian artery, with the right vertebral artery and right common carotid artery located adjacent to the right subclavian artery. The upper right image shows the location of the tear, and the lower right image demonstrates the dissection extending to the distal aorta.

Supplementary Figure 2 Preoperative Chest and Abdominal Vascular CTA Three-Dimensional Reconstruction. RSA: Right Subclavian Artery; RCCA: Right Common Carotid Artery; LCCA: Left Common Carotid Artery; LSA: Left Subclavian Artery; RVA: Right Vertebral Artery; LVA: Left Vertebral Artery.

Supplementary Figure 3 Intraoperative Anatomical Relationships of Blood Vessels. 1: Ascending aorta; 2: Brachiocephalic vein; 3: Left Common Carotid Artery; 4: Right Common Carotid Artery; 5: Right Vertebral Artery; 6: Right Subclavian Artery; 7: Left Subclavian Artery.

Supplementary Figure 4 Postoperative Chest and Abdominal Vascular CTA. The left side shows the postoperative relationships between the right subclavian artery, right vertebral artery, and right common carotid artery. The upper right image shows the proximal anastomosis, and the lower right image shows the thrombosed false lumen at the distal end.

Supplementary Figure 5 Postoperative Chest and Abdominal Vascular CTA Three-Dimensional Reconstruction.

Supplementary Video 1 Preoperative Chest and Abdominal Vascular CTA Three-Dimensional Reconstruction Dynamic Video.

Supplementary Video 2 Postoperative Chest and Abdominal Vascular CTA Three-Dimensional Reconstruction Dynamic Video.
